# Advances in Metal and Metal Oxide Nanomaterials for Topical Antimicrobial Applications: Insights and Future Perspectives

**DOI:** 10.3390/molecules29235551

**Published:** 2024-11-25

**Authors:** Belmina Saric Medic, Nikolina Tomic, Nefeli Lagopati, Maria Gazouli, Lejla Pojskic

**Affiliations:** 1 Laboratory for Human Genetics, Institute for Genetic Engineering and Biotechnology, University of Sarajevo, Zmaja od Bosne 8, 71000 Sarajevo, Bosnia and Herzegovina; belmina.saric@ingeb.unsa.ba (B.S.M.); nikolina.tomic@ingeb.unsa.ba (N.T.); lejla.pojskic@ingeb.unsa.ba (L.P.); 2 Laboratory of Biology, Department of Basic Medical Sciences, Medical School, National and Kapodistrian University of Athens, 75 Mikras Asias Str., 11527 Athens, Greece; nlagopati@med.uoa.gr; 3 2nd Department of Radiology, Medical Physics Unit, Medical School, National and Kapodistrian University of Athens, Attikon University Hospital, 1 Rimini Str., 12462 Athens, Greece

**Keywords:** metal nanoparticles, metal oxide nanoparticles, antimicrobials, topical application

## Abstract

Nanotechnology has seen significant growth in the past few decades, with the use of nanomaterials reaching a wide scale. Given that antimicrobial resistance is peaking, nanotechnology holds distinct potential in this area. This review discusses recent applications of metal and metal oxide nanoparticles as antibacterial, antifungal, and antiviral agents, particularly focusing on their topical applications and their role in chronic wound therapy. We explore their use in various forms, including coated, encapsulated, and incorporated in hydrogels or as complexes, proposing them as topical antimicrobials with promising properties. Some studies have shown that metal and metal oxide nanoparticles can exhibit cytotoxic and genotoxic effects, while others have found no such properties. These effects depend on factors such as nanoparticle size, shape, concentration, and other characteristics. It is essential to establish the dose or concentration associated with potential toxic effects and to investigate the severity of these effects to determine a threshold below which metal or metal oxide nanoparticles will not produce negative outcomes. Therefore, further research should focus on safety assessments, ensuring that metal and metal oxide nanoparticles can be safely used as therapeutics in biomedical sciences.

## 1. Introduction

During the last few decades, the development of nanotechnology and nanomaterials has grown significantly [1,2]. It is generally known that the nanoparticles (NPs) are defined as a distinct nano-object with all three Cartesian dimensions less than 100 nm. However, under a wider-range definition, a nano-object with just one of its characteristic dimensions in the range 1–100 nm is classified as a nanoparticle. Its other dimensions can be outside that range, but the lower limit of 1 nm is defined since atomic bond lengths are reached at 0.1 nm [3]. There are many types of nanoparticles, including fullerenes, carbon nanotubes, metal nanoparticles, metal oxide nanoparticles, liposomes, dendrimers, polymer nanoparticles, magnetic nanoparticles, micelles, and others [4]. The small size of nanoparticles allows them to easily penetrate human cells, facilitating their application not only in disease detection but also in targeted drug delivery [5].

Particular interest in the development of nanoparticles focuses on the development of nanoparticles of metals and metal oxides, due to their special characteristics, such as smaller particle size, large surface-to-volume ratio, and adjustable morphological properties. These features make them suitable for use in many fields, including microbiology, environmental protection, electronics, and biomedical sciences [1]. In biomedical applications, nanoparticles are used for disease diagnosis, for drug delivery, and as newly discovered drugs for the treatment of various diseases [6]. One of the most significant advancements in biomedical sciences is the use of nanoparticles for overcoming the limitations of traditional approaches to chemotherapy and radiotherapy in cancer treatment [2].

The most commonly explored nanoparticles include copper, silver, zinc, platinum, gold, and magnesium. Among metal oxides, the most studied nanoparticles are cerium oxide, zinc oxide, titanium oxide, copper oxide, and silver oxide. To enhance stability and biocompatibility, these nanoparticles are often coated or encapsulated with organic materials [5].

### 1.1. Nanoparticle Synthesis

The synthesis of nanoparticles can be carried out using physical, chemical, and biological methods, each with its own advantages and disadvantages. A fundamental classification of nanoparticle synthesis is into top-down and bottom-up approaches [7]. The most commonly used methods are chemical, such as organic and inorganic reduction of chemical materials, electrochemical methods, or chemical methods using irradiation. The least used methods for the synthesis of nanoparticles are physical, such as evaporation, condensation, laser ablation, etc. Nanoparticles obtained in these two ways pose a serious threat to the ecosystem regarding the use of toxic agents [7,8,9,10]. The main disadvantages of the top-down methods, such as mechanical milling and thermal and laser ablation, are changes in the surface chemistry and physicochemical properties, which leads to the impossibility of obtaining nanoparticles of very small sizes. To obtain nanoparticles of correct morphology, author Jamkhande and his collaborators provided contributions to methods, such as physical vapor deposition, chemical vapor deposition, sol gel methods, chemical reduction, hydrothermal method, solvothermal method, and many others [7]. They also highlighted the importance and advantages of biological synthesis. The biological method of nanoparticle synthesis, known as green synthesis, is increasingly preferred. This approach replaces traditional reducing agents and stabilizers with biological molecules produced by living systems, such as plants, bacteria, fungi, and algae [11]. The use of biological molecules reduces the toxicity of nanoparticles, making them practically safer for use. Compared to the other methods, green synthesis is much simpler, has higher cost-effectiveness, it is more efficient, and is environmentally friendly for the production of nanoparticles [8,9].

There are several methods of green synthesis of nanoparticles, namely, synthesis using enzymes, synthesis using vitamins, synthesis assisted by microwaves, and biologically based green synthesis. Biologically based green synthesis involves the use of bacteria, fungi, algae, plants, and phytochemicals [12]. Charbgoo and his collaborators in their work explained the advantages and disadvantages between different methods of green synthesis using the example of cerium oxide nanoparticles. According to them, plant-based green synthesis yielded spherical nanoceria with reduced toxicity and synthesized relatively inexpensive nanoceria, though sometimes this type of synthesis led to agglomerates’ formation. Mycosynthesis of nanoceria has advantages such as cost-effective synthesis in a shorter period, obtaining stable water-soluble particles with high fluorescent properties. Nutrient-mediated methods produced nontoxic nanoceria on human healthy cell lines, while biopolymer-based synthesis also led to forming smaller-size nanoceria with an insignificant cytotoxic effect on a healthy human cell line with a high degree of purity [13].

### 1.2. Characterization of Nanoparticles

The characterization of nanoparticles is a key step in their adoption for specific applications. Nanomaterials are characterized to explore various physical and chemical properties, and this process should involve multiple methods in order to capture all substantial characteristics [14]. Characterization is usually performed using various methods, such as UV-Vis spectroscopy, Fourier transform infrared spectroscopy (FT-IR), scanning electron microscope (SEM), transmission electron microscope (TEM), X-ray diffraction (XRD) [15], and other methods, such as inductively coupled plasma mass spectrometry (ICP-MS) [16]. Optical properties are analyzed by UV-Vis spectrophotometry. The FT-IR method is used to analyze the chemical bonds on the surface of the nanoparticle in order to analyze the presence of individual components. SEM analyzes the surface morphology, topography, and composition of nanoparticles, while TEM provides information on the exact size and shape of nanoparticles. The XRD methodology confirms the crystal structure, thus confirming the chemical nature of the components used for synthesis [17]. To characterize the stability and average size of nanoparticles, devices called “nanosizers” are used, which essentially measure the zeta potential [18]. The ICP-MS technique showed potential in nanomaterial characterization and quantification in the last few decades. It can provide information about nanoparticles and their features, such as size, concentrations, composition, amounts of proteins, etc. [16].

### 1.3. Antimicrobial Resistance

Intensive research in the field of nanotechnology by numerous scientists aims to address current global issues and crises, ultimately making life easier for humanity. One pressing problem today is the antimicrobial resistance (AMR). Shortly after the introduction of antibiotics into clinical use, pathogenic strains of bacteria appeared that were resistant to their effects. For example, after the introduction of penicillin in the treatment of soldiers’ wounds during the Second World War, penicillin-resistant strains of bacteria appeared. Already by the 1960s, this problem had reached pandemic proportions. Today, it poses a growing public health problem, with concerns that we are entering a so-called “post-antibiotic” era—an era reminiscent of the time before the discovery of antibiotic substances [19,20].

**Figure 1:**
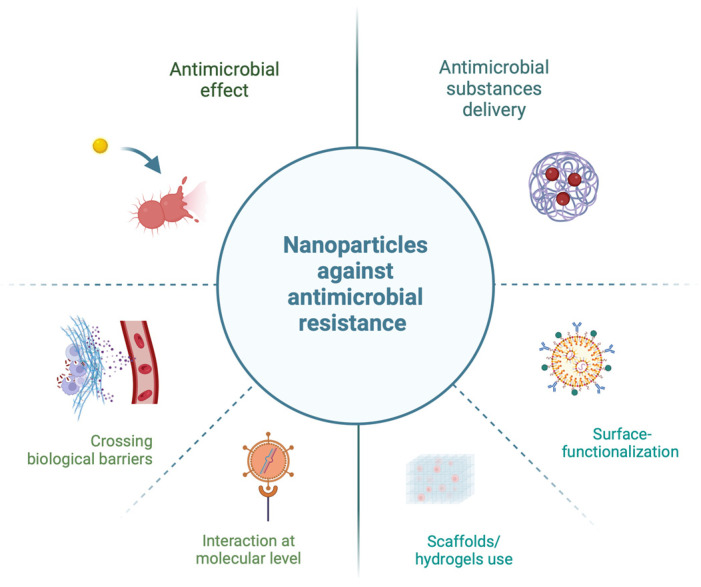
Nanoparticles’ potential for combating antimicrobial resistance (created at: https://BioRender.com).

Nanotechnology holds great potential for treating resistant strains of bacteria or fungi as well as for delivering substances with an inhibitory effect against them (Figure 1). The small size of nanoparticles allows them to interact with the biological systems at the molecular level, facilitating targeted drug delivery and enabling them to cross biological barriers, making them suitable for biological applications against microbes. Furthermore, different surface-functionalization methods (chemical, physical, or biological) can be used to specifically improve nanoparticles that are used to carry antimicrobials. This approach aims to achieve optimal effects through selective recognition, an improved payload-binding capacity, increased cellular uptake, and reduced cytotoxicity [21]. In recent studies, scaffolds/hydrogels are often used as 3D matrices to encapsulate them in order to improve stability and targeted antimicrobial drug delivery, with minimal risk of drug resistance development [22]. In addition, the efforts of the scientific and professional community have focused on the discovery of new antimicrobials, such as nanomaterials, but also on the adequate detection of genes for resistance and virulence. Traditional microbiological methods for detecting bacteria and bacterial resistance can be insufficient for rapid analysis, particularly for bacterial species that grow slowly in laboratory conditions or are non-cultivable. For these reasons, molecular genetic methods are a suitable choice for detecting pathogenic strains and identifying resistance and virulence factors [23].

In addition to molecular technologies, emerging problems require innovative solutions. Recent research has shown that artificial intelligence (AI) offers new prospects for combating antimicrobial resistance. AI, leveraging extensive data through machine learning and deep learning algorithms, can propose new antimicrobial agents, recommend effective treatments, identify resistance markers, optimize antibiotic selection, and improve delivery systems, including nanoparticle-based systems targeting the site of infection while minimizing toxicity. Researchers have already attempted to use convolutional neural networks to identify drug-resistant bacterial cells using TEM images. However, challenges remain in implementing AI for treating antimicrobial resistance, primarily due to the lack of a large, standardized dataset for analysis [24].

## 2. Metal Nanoparticles and Metal Oxide Nanoparticles’ Biological Activities and Utilization

**Figure 2:**
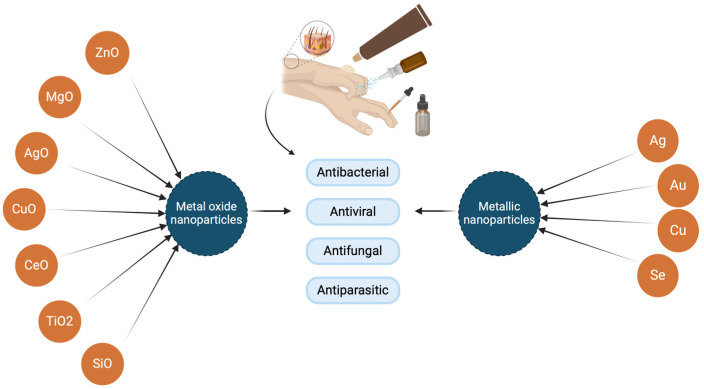
Schematic representation of the most researched nanoparticles of metals and metal oxides with antimicrobial activity for potential in topical use (created at: https://BioRender.com).

Metal and metal oxide nanoparticles exhibit pronounced antiviral, antibacterial, antiparasitic, and antifungal effects (Figure 2). Additionally, they have a potential therapeutic effect, as they can facilitate the controlled release of the drug, but also the delivery of the drug to the site of infection, which ultimately increases the clinical effect [25]. For this reason, nanoparticles could serve as potential systems for the delivery of antimicrobials, so besides controlled drug delivery and targeted delivery, these nanoparticles have functional bioavailability as well as low toxicity [26]. Metal nanoparticles and metal oxide nanoparticles have the ability to create non-specific microbial resistance pathways that not only inhibit bacterial growth but also enable a greater range of antimicrobial activity [27]. The mechanisms of antimicrobial activity of nanoparticles and real-life toxicity are not well understood [28]. Their action is not specific, making it difficult to explain their antimicrobial effects through a single mechanism [29]. Currently proposed mechanisms are metal ion release, induction of oxidative stress, and non-oxidative mechanisms. Several synchronous mechanisms of action against microorganisms imply several simultaneous mutations in the bacterial cell to develop antimicrobial resistance, and for that reason, it is hard for bacteria to become resistant to metal NPs [28].

One of the basic mechanisms of the antimicrobial effect of metal nanoparticles and metal oxides is the production of ROS molecules, where they exert their potentially toxic effect and, among other things, given their size, have the ability to penetrate organs and accumulate them. Considering that, the control of the production of ROS molecules and the toxic effect of nanoparticles depends on many factors and properties, such as size, shape, morphology, composition, solubility, aggregation, and particle uptake [30]. In the study of Alarcon et al., silver nanoparticles penetrated to certain organs of the mice, more specifically the liver, kidneys, and spleen, although they were used for a topical application in tissue engineering. However, the research itself points to silver accumulation primarily within the surrounding tissue [31].

### 2.1. Antibacterial Action of Nanoparticles of Metals and Metal Oxides and Their Effects in Combination with Antibiotics and Herbal Preparations for Potential Topical Application

As already mentioned, metal and metal oxide nanoparticles are well known for their antimicrobial activity. In this review, it will be discussed mainly regarding their utilization as topicals against a wide spectrum of microbes. Silver nanoparticles have been used as antibacterial agents as colloidal silver. Gold nanoparticles have antimicrobial properties against Gram-negative and Gram-positive bacteria, while having low toxicity and great absorption properties. Zinc and zinc oxide nanoparticles are biocompatible antimicrobials against bacteria, such as *Campylobacter jejuni*, *Salmonella typhimurium*, *Klebsiella pneumoniae*, and *Neisseria gonorrhoeae*, considering that zinc has numerous biological roles in the human body. Titanium and titanium dioxide nanoparticles are known for their inhibition of bacterial growth, which can be used for infection control. Besides these well-known metal and metal oxide nanoparticles as antibacterials, there are cerium and cerium oxide nanoparticles, iron and iron oxide nanoparticles, known for their antibiofilm potential, copper and copper oxide nanoparticles, and many others [32].

**Figure 3:**
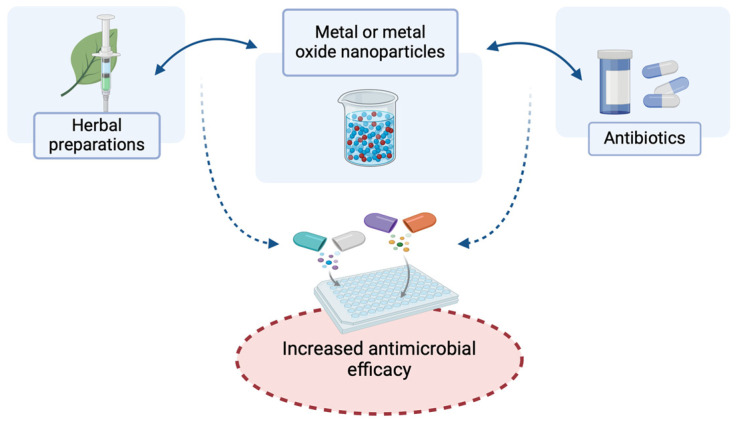
Synergistic enhancement of the antimicrobial effect through a combination of metal and metal oxide nanoparticles with antibiotics and herbal preparation (created at: https://BioRender.com).

Many studies have shown that the antibacterial effectiveness of numerous antibiotics increases when the same antibiotics are in complex with metal nanoparticles (Figure 3). For example, the results of research of the synergistic antibacterial effect of silver nanoparticles (synthesized with the water extract of *Ulva fasciata* algae) and numerous antibiotics, such as chloramphenicol, fosfomycin, cefuroxime, cefotaxime, and azithromycin, against bacteria, such as *Escherichia coli* and *Salmonella* spp., showed that the antibacterial effect is enhanced by the presence of silver nanoparticles compared to a single control antibiotic [33].

Also, zinc oxide nanoparticles in a complex with methicillin had an antibacterial effect on *Staphylococcus aureus* species [34], while zinc oxide nanoparticles in complex with ampicillin had an inhibitory effect on *Klebsiella pneumoniae* by activating ROS molecules (reactive oxidative species) and rupturing the cell wall [35]. Some research showed that nitric oxide donors can act as antimicrobial enhancers when they are combined with ZnO NPs, where they exhibited synergistic properties. Nitric oxide donors, as well as ZnO NPs, did not show toxic effects against mammalian cells. In one study, a nitric-oxide-releasing polymer that had a topcoat of zinc oxide nanoparticles showed antimicrobial activity against *S. aureus* and *P. aeuroginosa* [36].

Synergistic action resulting in increased antibacterial potential has become one of the characteristics of the combination of metal nanoparticles and their oxides with antibiotics [37].

Biosynthesized colloidal ZnO nanoparticles were employed, together with cetylpyridinium chloride and chlorhexidine gluconate, into a topical, cosmetic, and dermatological skin cream to test its antimicrobial efficacy on five different clinical pathogens, such as *S. aureus*, *E. coli*, *Pseudomonas aeruginosa*, *Salmonella typhi*, and *Shigellam*, while their antimicrobial effect was confirmed in vitro [38].

Research by Wronska et al. indicated that, in addition to the antimicrobial effect in the antibiotic–nanoparticle complex, the antibacterial effect was enhanced between the nanoparticle complex and phytochemicals. They found that combined therapy using silver nanoparticles encapsulated with ursolic acid exhibited a synergistic effect, demonstrating bactericidal activity against *E. coli* and *P. aeruginosa*. The joint or co-delivery of this complex significantly reduced the growth of *S. aureus* strains. However, the effect was weakest against *Staphylococcus epidermidis* [39].

Moreover, Mujahid and his collaborators confirmed the successful use of metal nanoparticles and metal oxide nanoparticles as potential drug delivery systems, considering their antimicrobial, antioxidant, and anticancer effects. The mechanism of their action affects the fact that antibacterial resistance has been successfully overcome, whereby formation of biofilms was inactivated [40].

### 2.2. Metal and Metal Oxide Nanoparticles as Topical Antifungals

Besides antibacterial, nanoparticles of metal and metal oxide also showed antifungal properties. Metal (Ag, Au, Cu, Pd, and Se) and metal oxide (AgO, CuO, MgO, TiO_2_, and ZnO) nanoparticles are well known for their antifungal activity (Table 1). This kind of action has been investigated in multiple studies against a large number of fungi, such as *Candida* spp., *Fusarium* spp., *Aspergillus* spp., *Rhizopus* spp., *Rhizoctonia* spp., *Trichoderma* spp. and many others. These nanoparticles were either combined with antifungals or they were doped. Some studies showed that combining nanoparticles, such as AgNPs, can result in synergistic effects. Also, nanoparticles enclosed or coated with antifungals can result in increasing mechanical damage, whereby when they are enclosed with antifungals rather than coated, it can reduce their toxicity. Mechanisms of their antifungal action showed that these nanoparticles can affect fungi in a way that damages their cell wall, DNA, hyphae, and spores, generating ROS molecules or releasing ions, or even that they can have antibiofilm activity [41]. AgNPs are well known for their antibacterial as well as antifungal properties. Also, they have been used as carriers for antifungals, such as nystatin and voriconazole [42]. AgNPs had great antifungal effects against the widest spectrum of fungi, including species from genera such as *Candida*, *Malasssezia*, *Cryptococcus*, *Monilia*, *Fusarium*, *Cladosporium*, *Trichophyton*, *Mortierella*, *Aspergillus*, *Stachybotrys*, *Penicillium*, and many others [43]. Moreover, silver nanoparticles (Nivasha spray 15 ppm) had more effectiveness compared to clotrimazole 1% on candida vaginitis in a randomized controlled clinical trial and were suggested as an alternative drug [44].

Additionally, gold nanoparticles (AuNPs) were also used as carriers for antifungals. AuNPs’ mechanism is based on mitochondrial calcium homeostasis disturbance of *C. albicans*. Gold nanoparticles have been examined for their application against the following fungi: *Candida tropicalis*, *C. albicans*, *C. glabrata*, *C. parapsilosis*, *Issatchenkia orientalis* (*C. krusei*), and *Fusarium oxysporum* [43].

For biomedical purposes, metal and metal oxide nanoparticles can be used as topical antifungals [42].

The study of Eremenko et al. showed that cotton fabrics impregnated with silver and binary silver/copper nanoparticles had antifungal and antibacterial effects, even six months after multiple washing or ironing [45].

Another study showed great potential of gel-based rutin-AgNPs against *C. albicans*. These types of nanoparticles could be used as topical antifungals and as their carrier for wound healing. These type of NPs were synthesized using gum acacia [46]. In the study of Abdallah and Ali, green biosynthesized EG-AgNPs (*Erodium glaucophyllum*) showed a strong inhibitory effect against *C. albicans* and as a topical antifungal for oral candidiasis in an in vivo mouse model [47].

Regarding the use of metal oxide nanoparticles as antifungals, zinc oxide nanoparticles (ZnONPs) coated with chitosan-linoleic acid showed an antifungal effect against *C. albicans* with antibiofilm potential [48]. As well as their action against *C. albicans*, they showed antifungal activity against *Aspergillus niger*, *Penicillium expansum*, *Fusarium solani*, *Aspergillus fumigatus*, and *Fusarium graminearum* [43]. Also, a similar effect against *C. albicans* and its biofilm formation was shown by titanium oxide nanoparticles [49]. In vitro and in vivo studies showed that titanium oxide nanoparticles had little or no toxic effects in some studies, while some studies showed that these types of nanoparticles could have genotoxic or cytotoxic effects [43].

Iron oxide nanoparticles, which were originally approved by the FDA (Food and Drug Administration) for iron deficiency, in the study of Liu et al. showed potential for oral usage in the topical treatment of dental caries, whereby *C. albicans* was found to also be a dental-caries-associated pathogen [50]. Iron oxides nanoparticles showed great antifungal potential against the following fungi: *Mucor piriformis*, *A. niger*, *C. parapsilosis*, *C. albicans*, *Penicillium chrysogenum*, *C. tropicalis*, *C. glabrata*, *Alternaria alternata*, *Cladosporium herbarum*, and *Trichothecium roseum*. Copper and copper oxide nanoparticles have been noticed for their suppressive effect against *C. albicans*, *Cladosporium herbarum*, and other types of yeast. What is most important, copper is known as a nontoxic heavy metal [43].

## 3. Metal and Metal Oxide Nanoparticles in the Treatment of Chronic Wound Infections

Recent research in the field of nanotechnology indicated the potential successful use of various nanoparticles in the treatment of chronic wounds, especially in complexes of inorganic and organic materials [51,52]. Furthermore, metal and metal oxide nanoparticles have been known for their use in dermatology and cosmetics as antimicrobial protection to the skin. Beside their usage for wound healing, metal and metal oxide NPs have been used for treatment of dermatological infections. Notably, silver and gold nanoparticles are increasingly promoted as novel cosmeceuticals, including their use in anti-perspiration sprays [53].

### 3.1. Basic Characteristics of Chronic Wounds

The term ‘chronic wounds’ refers to wounds that have not healed within three months, and includes arterial, venous, diabetic ulcers/wounds, and decubitus ulcers. Venous ulcers affect 1–2% of the adult population, with a higher prevalence in the elderly and women [54]. They arise as a result of venous hypertension and congestion (clogging) due to venous thrombosis, as a result of which red blood cells and macromolecules leak into the perivascular space [55,56]. Venous ulcers are mostly shallow, but cover a larger area of the skin, with irregular edges, mostly affecting parts of the lower extremities [54]. Arterial ulcers are rare and arise as a result of arterial insufficiency due to atherosclerosis or thromboembolism [57]. Arterial ulcers most often occur on bony protrusions, while the therapy of such wounds may require restoration of peripheral blood flow by angioplasty or reconstructive surgery [58,59]. Diabetes mellitus is one of the leading causes of death in the world [60], and diabetic foot ulcers are actually a common and very dangerous complication of this disease [61]. Peripheral neuropathy associated with this disease leads to weakening of foot structures as well as a loss of sensation [54]. Repetitive mechanical stress with disruption of blood and oxygen flow and metabolic disorders associated with hyperglycemia lead to the accumulation of inflammatory factors that induce oxidative stress, damaging the skin and impairing its function [61,62,63]. Such outcomes of diabetes with ulceration can lead to limb amputation and death [60]. Decubitus is common in immobile and paralyzed patients. It is necessary to periodically turn the patient and change their position, since due to long-term pressure on the surface of the skin, ischemia may occur due to compression of the capillaries [54]. Tissue hypoxia and ischemia lead to injury and, ultimately, result in necrosis [64].

### 3.2. Main Factors for Chronic Wound Formation

**Figure 4:**
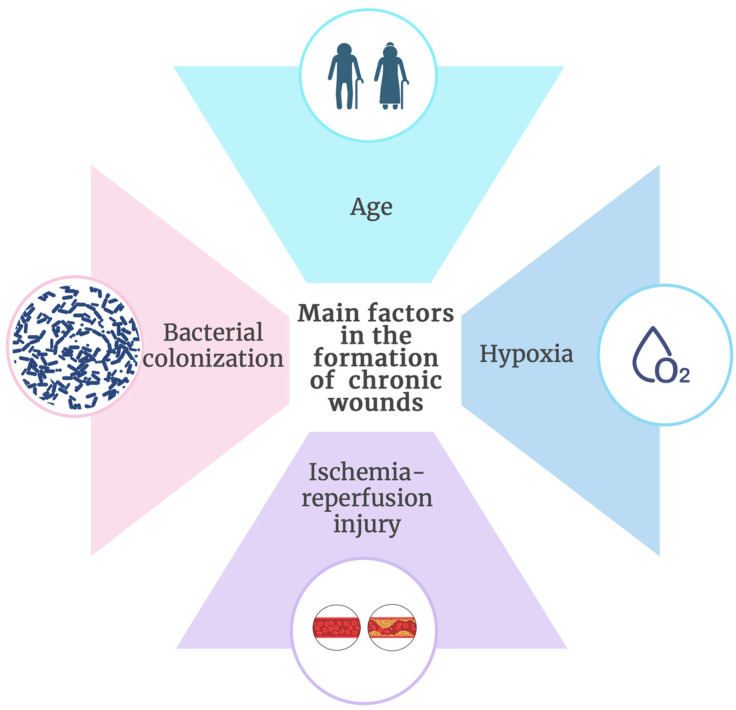
Main factors in the formation of chronic wounds (created at: https://BioRender.com).

Age, hypoxia, ischemia-reperfusion injury, and bacterial colonization are among the main factors in the formation of chronic wounds (Figure 4) [65,66].

In addition to direct damage to the host cell, bacteria attract leukocytes, resulting in an increase in inflammatory cytokines, proteases, and ROS molecules, whereby this cascade leads to cellular damage, thus preventing skin regeneration and wound healing [55,66]. As one of the most common causes and factors for the transformation of an ordinary wound into a chronic one, the existence of bacterial colonization or infection, which lead to long-term treatment, is cited [67,68]. The most common pathogens of chronic wounds are *S. aureus*, *P. aeruginosa*, and β-hemolytic streptococci [69]. Besides *S. aureus* and *P. aeruginosa*, which are the most frequently isolated bacteria from chronic wounds, some of the other characteristic microorganisms that colonize these wounds are *Enterococcus* spp., *Acinetobacter* spp., then *C. albicans* and *Aspergillus* spp. In addition, some of the listed pathogens are also known as multi-resistant strains, which today represents one of the main growing public health problems at the global level [70]. Then, there may be other bacteria, such as *E. coli*, *Enterobacter cloacae*, *Klebsiella* spp., *Streptococcus* spp., *Proteus* spp. and numerous others [71].

Bacteria that colonize chronic wounds mostly form polymicrobial biofilms, where in fact 60% of all chronic wounds are colonized by bacteria that form biofilms [68]. Biofilms provide optimal conditions for the bacteria, which enables their settlement and survival and synergistic action between strains, while the host’s immune response and antibacterial response lag behind. Biofilms are essentially a physical barrier that prevents macrophages and neutrophils from destroying bacteria [72]. In addition, hypoxia accompanying chronic wounds greatly contributes to bacterial colonization [55,65].

### 3.3. The Most Frequently Used Nanoparticles of Metals and Metal Oxides in the Treatment of Chronic Wounds

In terms of intrinsic factors related to catalytic, optical, and melting properties, nanoparticles of metals and metal oxides, unlike traditional preparations, have numerous advantages in the treatment of wounds [73], especially due to their characteristics, such as size, porosity, metal resistance to decomposition in aqueous solutions, and numerous surface properties [74,75]. The most commonly used metal and metal oxide nanoparticles in wound treatment, along with their main features, are summarized in Table 2.

AgNPs are very effective for topical application of drugs in wound healing due to their high surface-area-to-volume ratio. Silver nanoparticles have a weaker binding affinity to the surface, and for this reason, the use of hydrogels, i.e., 3D structures in which the nanoparticles are incorporated, was proposed for their application in the treatment of wounds. This ultimately enabled nanoparticle retention, targeted delivery, as well as controlled drug release [76]. There are many types of hydrogels, such as chitosan-based nanofibrous membranes, zwitterion polymers, lignin-based hydrogels, impregnated chitosan PEG hydrogels, and many others [11]. Oleogels containing silica–silver nanomaterials demonstrated potent antimicrobial activity with low or no cytotoxic effects [77]. Also, silver nanoparticles incorporated into hydrogels had antibiofilm properties [78,79], showing their potential use in the treatment of resistant forms of bacterial strains that form biofilms in the oral cavity or chronic wounds. Given that AgNPs can cross the blood–brain and blood–testis barriers [80], and in higher concentrations can induce cell necrosis and cell membrane rupture [81], many authors recommend the use of silver nanoparticles in the form of topical preparations for the treatment of wounds, i.e., incorporated in polyacrylamide gels [82]. Thus, for example, in order to prevent post-operative infection during implant placement operations, silver-coated implants are used, and there are other commercially available products based on silver nanotechnology that have found use in other industries, such as textiles and food [83,84,85].

It is known that silver nanoparticles have been used for infection control and wound dressing for decades [86]. However, due to toxicity issues, there is an increasing focus on ZnO [87], cerium oxide nanoparticles [6], and TiO_2_ nanoparticles [88].

ZnO nanoparticles are used for the treatment of slow-healing wounds, due to their bacteriostatic and bactericidal effect, and the possibility of promoting collagen synthesis [74,85,89,90]. Zinc essentially regulates enzymes, such as DNA and RNA polymerase, ribonuclease, and thymidine kinase, which ultimately leads to the regulation of burns and slow-healing wounds [90]. ZnO nanoparticles showed lower toxicity and greater permeability through membranes compared to silver and gold nanoparticles [89], while chitosan nanofibers with ZnO nanoparticles and dual antibiotics exhibited synergistic antibacterial and wound healing effects and also potentially minimized cytotoxicity compared to individual antibiotics [91]. However, due to the intrinsic toxicity of these nanoparticles, they still require further research in the treatment of chronic wounds and infections.

Cerium oxide nanoparticles, also known as nanoceria, have multiple biological activities, where, very similar to silver nanoparticles, they can act as antibacterial, anticancer, and antioxidant agents [1,6]. Nanoceria can be used for drug delivery but have also shown antiparasitic activity [6]. Nanoceria showed significant antibacterial activity against both Gram-positive and Gram-negative bacteria. Special importance is placed on their bactericidal effect on highly resistant types of bacteria, such as *S. aureus*, *E. coli*, *P. aeruginosa*, *K. pneumonia*, and others [4,92]. The antibacterial effect is manifested differently depending on the concentration and pH value of the medium [93]. Although the mechanism of action itself is not yet precisely explained, it is believed that the bactericidal effect of nanoceria is based on the creation of a large number of ROS molecules and free radicals [6]. Research of Pulido-Reyes et al. came to the conclusion that the toxicity of nanoceria does not depend on the concentration, surface charge, or size, but toxicity depends on the percentage of surface Ce^3+^ [94]. However, there is no clear knowledge about the impact of the application of green synthesis on the percentage of Ce^3+^ surface area on cerium oxide nanoparticles, and this remains to be investigated [13]. Also, nanoceria have been used as therapeutic agents for tissue restoration and regeneration, due to the possession of angiogenic properties, whereby nanoceria also have the ability to induce tissue renewal [95]. In addition, nanoceria showed antidiabetic activity [96]. In vitro studies by scientists Zhao and colleagues showed that dressings based on the principle of cerium oxide nanoparticles incorporated into alginate hydrogels had significant antioxidant, biocompatible, and hemocompatible properties. The animal studies of these scientists on the stubborn skin of rats showed that bandages such as cerium oxide alginate hydrogels accelerated the wound-healing process [97]. The study by Luo et al. investigated the use of poly(2-hydroxyethyl methacrylate)-cytosan hydrogels with incorporated nanoceria in the treatment of chronic wounds. The research results showed a successful antibacterial effect on *S. aureus* and *E. coli*, as well as antibiofilm potential. It is interesting that these types of hydrogels had no toxic effect on cell viability. Ultimately, their research results indicated the possibility of using these gels in the treatment of chronic wounds in the form of dressings. However, high concentration and long exposure can lead to toxicity and, therefore, it is necessary to investigate the mechanism of action, and to optimize the effect in different types of wounds and populations, which implies clinical research as well as approval by regulatory authorities [98].

TiO_2_ NPs have various applications: they are used for paints, plastics, rubber, and energy storage, in cosmetics (sunscreens), and for drug delivery and in food additives [99]. Lately, there has been an increased tendency to use pure or coated titanium dioxide nanoparticles in the cosmetic and pharmaceutical industries.

In the context of use in the treatment of chronic wounds, they are often incorporated with hydrogels, e.g., Ag-loaded TiO_2_ nanorods with a gellan gum biopolymer hydrogel film (Ag@TiO_2_NRs/GG) were reported to lead to 100% wound healing on Sprague Dawley rats after 14 days of treatment. Skin tissue regeneration was demonstrated through clear dermis, a thicker subcutis layer, and the epidermis [83]. Another similar complex has recently been engineered for enhanced antibacterial and wound-healing efficacy—titanium–hydroxyapatite nanocomposite hydrogel (TiO_2_–HAp@PF-127@CBM). Jing et al. concluded that its dressing’s capacity to enhance granulation tissue formation and support epidermal regeneration highlights its potential for antibacterial effects and wound-healing applications [100].

**Table 2:** Most commonly used metal and metal oxide nanoparticles in wound treatment and their properties.

Nanoparticles	Active Formation	Activity	References
Silver (AgNPs)		Antimicrobial	[76,77,79,80,81,82,83,85,86]
	Incorporated in hydrogels	Antibiofilm	
		Healing properties	
Zinc oxide (ZnO NPs)	Dressings	Bacteriostatic	[74,85,89,90,91]
	Incorporated in hydrogels	Bactericidal Collagen promoting	
Cerium oxide (CeO NPs)		Antibacterial	[1,6,92,93,94,95,96,97,98]
	Dressings	Antibiofilm	
	Incorporated in hydrogels	Antioxidant	
		Angiogenic	
Titanium dioxide (TiO_2_ NPs)	Incorporated in hydrogels	Antimicrobial	[93,99,100]
	Nanorods	Healing properties	

## 4. Use of Metal and Metal Oxide Nanoparticles in the Antiviral Treatment

Metal and metal oxide nanomaterials are used as effective antiviral agents. These nanomaterials can be of different sizes, shapes, and charges, whereby their antiviral effect can be manifested in the form of aqueous dispersions or incorporated into other materials, such as coating materials or packaging materials [101].

In general, the basic mechanisms of antiviral action of nanomaterials of metals and metal oxides are as follows [102,103,104,105,106,107,108,109]:

1. Binding of nanoparticles to the surface structures of viral particles, which ultimately prevents viral binding to the host cell.
2. Generation of reactive oxygen species (ROS), leading to the denaturation of viral macromolecules (proteins, nucleic acids, and lipids).
3. Inactivation of viral glycoproteins through the destruction of disulfide bonds between proteins.

### The Most Known Metal and Metal Oxide Nanoparticles in Antiviral Treatment

According to the reference literature, nanoparticles of silver, gold, zinc, copper, titanium, and iron and their oxides have antiviral activity (Table 3) [101,110,111,112]. In the context of the recent pandemic caused by severe acute respiratory syndrome coronavirus *2* (SARS-CoV-2), silver nanoparticles showed a significant antiviral effect with concentrations in range of 1 to 10 ppm, while concentrations above 20 ppm had a cytotoxic effect [113]. Other studies have reported that different sizes of silver nanoparticles showed antiviral effects against multiple other viruses, such as influenza virus, HBV (hepatitis B virus), and HSV-1 (herpes simplex virus type 1). In the study of Orlowski et al., AgNPs synthesized using tannin acid showed an antiviral effect against HSV-1, and considering that HSV-1 and HSV-2 have similar protein structures, these AgNPs could be used as topicals to treat HSV-2 infections of the anogenital area and oral herpes infections [114]. Beside this study, the study of Tavakoli et al. also showed the potency of using copper oxide nanoparticles for treatment of genital or orolabial HSV infections, using it in different types of formulations [110]. Recently, the antiviral effects of the thermally expanded graphite (TEG)–copper oxide (CuO) nanocomposite against HSV-1 have been investigated. It was demonstrated that it could decrease the viral load and affect viral replication in a dose-dependent manner [115].

Despite the promising prospects for antiviral applications and therapies highlighted by numerous in vitro and in vivo studies using metal nanoparticles and metal oxides, further investigation into the potential toxicity of these nanoparticles in humans and animals is essential. It is important to examine and determine the various factors influencing their action, as many of these nanoparticles can interact with other biological structures, particularly tissues, based on their charge [101].

## 5. Conclusions

Due to the emergence of an increasing number of resistant strains of microorganisms to antibiotics, antimicrobial resistance today represents a global health problem that is not decreasing. According to the data of the World Health Organization, the number of people who are dying due to a direct connection with antibacterial resistance is increasing, and the so-called multi-resistant bacterial strains are rising, which ultimately leads to the fact that we are entering the “post-antibiotic” era, that is, an era without antibiotics. Global experts have made efforts to slow this growing problem through various guidelines, prompting a significant redirection of research toward identifying new potential agents and materials for therapeutic use.

In this context, nanomaterials show great potential in eradicating microbes since they have shown their promising antibacterial, antiviral, and antifungal effects in numerous in vivo and in vitro studies. This antimicrobial effect especially refers to the nanoparticles of metals and metal oxides due to their unique characteristics. Consequently, these nanoparticles can be utilized not only for the treatment of various diseases but also for drug delivery.

Recent research has shown that the antibiotic effect is increased when antibiotics are combined with metal or metal oxide nanoparticles, which also indicated their synergistic effect. In addition, complexes of other potential antimicrobial substances and nanoparticles have this effect. Such complexes, especially when incorporated into materials such as hydrogels, hold particular importance.

Currently, there is a wide range of commercially available antibiotic complexes, antimicrobial substances, metal and metal oxide nanoparticles, hydrogels, and other biological systems that are successfully used in biomedical sciences, as well as in the textile and food industries. However, given the potential toxicity of metal and metal oxide nanoparticles, further research should prioritize safety testing. This includes monitoring the behavior of nanoparticles within organisms and understanding their mechanisms of action to ensure their safe use in therapies for humans, animals, and plants.

## Figures and Tables

**Table 1:** Literature data on the antifungal effects of metal nanoparticles and metal oxides on individual fungi.

Nanoparticles	Susceptible Species	References
Silver (AgNPs)	*Candida*, *Malasssezia*, *Cryptococcus*, *Monilia*, *Fusarium*, *Cladosporium*, *Trichophyton*, *Mortierella*, *Aspergillus*, *Stachybotrys*, *Penicillium*	[42,43,44,45,46,47]
Gold (AuNPs)	*Candida tropicalis*, *C. albicans*, *C.glabrata*, *C. parapsilosis*, *Issatchenkia orientalis (C. krusei)*, *Fusarium oxysporum*	[43]
Zinc oxide (ZnO NPs)	*Candida albicans*, *Aspergillus niger*, *Penicillium expansum*, *Fusarium solani*, *Aspergillus fumigatus*, *Fusarium graminearum*	[43,48]
Titanium dioxide (TiO_2_ NPs)	*C. albicans*	[43,49]
Copper and copper oxide	*C. albicans*, *Cladosporium herbarum*	[43]
Iron oxide	*C. albicans*, *Mucor piriformis*, *A. niger*, *C. parapsilosis*, *C. albicans*, *Penicillium chrysogenum*, *C. tropicalis*, *C. glabrata*, *Alternaria alternata*, *Cladosporium herbarum*, *Trichothecium roseum*	[50]

**Table 3:** Summarized antiviral effects of metal nanoparticles as potential topicals.

Nanoparticles	Affected Virus	References
AgNPs	SARS-CoV-2	[113]
AgNPs	HBV	[114]
AgNPs	HSV-1 and HSV-2	[114]
CuNPs	HSV	[110,115]

## Data Availability

There are no data associated with this publication.
